# Evolutionary Conservation of Bacterial Essential Metabolic Genes across All Bacterial Culture Media

**DOI:** 10.1371/journal.pone.0123785

**Published:** 2015-04-20

**Authors:** Oren Ish-Am, David M. Kristensen, Eytan Ruppin

**Affiliations:** 1 The Blavatnik School of Computer Science, Tel Aviv University, Tel Aviv, Israel; 2 National Center for Biotechnology Information, National Library of Medicine, National Institutes of Health, Bethesda, Maryland, United States of America; 3 The Sackler School of Medicine, Tel Aviv University, Tel Aviv, Israel; 4 Dept. of Computer Science and the Center for Bioinformatics & Computational Biology, the University of Maryland, Maryland, United States of America; Centre for Cellular and Molecular Biology, INDIA

## Abstract

One of the basic postulates of molecular evolution is that functionally important genes should evolve slower than genes of lesser significance. Essential genes, whose knockout leads to a lethal phenotype are considered of high functional importance, yet whether they are truly more conserved than nonessential genes has been the topic of much debate, fuelled by a host of contradictory findings. Here we conduct the first large-scale study utilizing genome-scale metabolic modeling and spanning many bacterial species, which aims to answer this question. Using the novel *Media Variation Analysis*, we examine the range of conservation of essential vs. nonessential metabolic genes in a given species *across all possible media*. We are thus able to obtain for the first time, exact upper and lower bounds on the levels of differential conservation of essential genes for each of the species studied. The results show that bacteria do exhibit an overall tendency for differential conservation of their essential genes vs. their non-essential ones, yet this tendency is highly variable across species. We show that the model bacterium *E*. *coli* K12 may or may not exhibit differential conservation of essential genes depending on its growth medium, shedding light on previous experimental studies showing opposite trends.

## Introduction

A gene can be classified as *essential* or *nonessential*, depending on its effect on an organism's fitness [1]. It is considered essential if its knockout results in a lethal phenotype and nonessential if the knocked-out organism is viable. Almost four decades ago, Alan Wilson and colleagues proposed that the genetic rate of evolution should be dependent on gene importance, i.e., essential genes should evolve more slowly than nonessential genes [2]. This has been termed the *knockout-rate* (KOR) *hypothesis*, linking between gene functional indispensability and rate of evolution [3]. Since the publication of the KOR hypothesis, extensive research has been devoted to measuring whether essential genes are in fact more evolutionary conserved than nonessential genes, with equivocal results.

In eukaryotes, most studies seem to refute the KOR hypothesis: Hurst and Smith measured the substitution rates between mouse and rat orthologous genes [3]. They also surveyed the knockout phenotypes of 175 mouse genes, and concluded that there was no difference between the evolutionary rates of essential and nonessential genes. Hirsh and Fraser observed that essential genes do not evolve slower than nonessential genes in *S*. *cerevisiae*. Nevertheless, when analyzing the evolutionary distances between the genes of *S*. *cerevisiae* and *C*. *elegans* they found a weak negative correlation between gene importance and evolutionary rate [4]. Pál and Hurst analyzed protein substitution rates using three close relatives of *S*. *cerevisiae* and concluded that it was gene expression levels and *not* essentiality that were responsible for the small effect of dispensability on protein substitution rates [5]. Consequently, using a different methodology to identify orthologs and a different set of transcriptome data, Hirsh and Fraser confirmed their previous conclusions that a significant correlation between dispensability and evolutionary rate exists, even when controlled for expression levels [6]. Working with *S*. *cerevisiae*, Zhang and He showed that protein evolutionary rate was significantly correlated to dispensability, even when controlling for gene expression levels and excluding duplicate genes [7]. Wang and Zhang studied S. cerevisiae, claiming that the weakness of the correlation between gene importance (determined by the reduction in growth observed after the knockout of a gene) and evolutionary rate is factual, and does not result from the disparate data sources and analysis methods used by the previous studies [8].

In the bacterial domain, opinions also vary: Relying on an experimental essentiality database, Jordan *et al*. found that the KOR hypothesis holds for *E*. *coli* and also, albeit to a lesser degree, for *Helicobacter pylori* and *Neisseria meningitidis* (inferring essentiality by homology to *E*. *coli*) [1]. Rocha and Danchin claimed that gene expression levels were markedly more important than gene essentiality in constraining amino acid substitution rates in *E*. *coli* and *Bacillus subtilis* [9]. In line with Jordan *et al*., Gong *et al*. found *E*. *coli* essential genes to be significantly more evolutionary conserved than nonessential genes [10]. Focusing on *Pseudomonas aeruginosa*, Dötsch *et al*. found a significant correlation between gene essentiality and evolutionary conservation, a correlation which, though weakened when gene expression was accounted for, remained significant [11].

A central caveat for most of the studies reviewed above is that they did not determine evolutionary conservation rates and gene importance on the same growth media: Evolutionary conservation rates were estimated by comparing the genes of related species that evolved on unknown historic media. Gene importance was determined according to gene essentiality experiments that were carried out on laboratory media. The extent to which lab media reflect the organisms' historic habitats is unknown and becomes critical when studying phenomena that are strongly media dependent such as gene essentiality [3], [12]. To circumvent this problem we conduct a genome scale metabolic modeling (GSMM) investigation to determine metabolic gene essentiality in a media-independent manner. GSMMs are built around a stoichiometric matrix S, formed from the stoichiometric coefficients of the reactions comprising the metabolic network, and an accompanying genes to proteins to reactions (GRP) mapping. Given such a GSMM, constraint-based modeling (CBM) analysis methods assume a metabolic steady-state under which feasible flux distributions satisfy a stoichiometric mass-balance requirement, thermodynamic constraints, and constraints on enzymes’ capacities. By imposing a set of governing cellular constraints, the behavior of the network can be described by the flux activity of its metabolic reactions.

In recent years, numerous CBM analysis methods have been developed that yield fairly accurate predictions of key metabolic phenotypes such as growth rate, nutrient uptake rates and gene essentiality [13]. Metabolic models along with such methods, have been extensively employed in the analysis of bacterial metabolism, successfully addressing both basic science and applied goals [14]–[20]. Metabolic model contributions include drug discovery [21], testing biological hypotheses [22], understanding network robustness [23] and metabolic engineering tasks [24]. Flux Balance Analysis (FBA) is currently the most widely studied CBM method, which relies on linear programming to search for an optimal steady state solution that maximizes a certain objective function (e.g. growth rate or metabolic yield) among all feasible steady state solutions [25].

This study employs GSMM to systematically study the differential evolutionary conservation of essential vs. nonessential metabolic genes in bacterial genomes. We present a novel computational approach—Media Variation Analysis (MVA), which enables us to tackle this long standing question in a media-independent manner, bypassing the potential bias introduced by using gene essentiality determined on lab media. Unless stated otherwise, all media referred to in this paper are *in-silico* media—a certain subset of the metabolic model intake reactions and should not be confused with laboratory media. To conduct a large scale study across many species, automatically generated models from the SEED project [26] were utilized in addition to existing human-curated models. Overall, this study covers 58 bacteria species using 69 metabolic models, where some species have both automated and human curated models.

## New Approaches

We present two new computational approaches for identifying gene essentiality across *in-silico* media—*Media Variation Analysis (MVA)* and *Essential Gene Sets (EGS)* analysis. Here we present a brief overview of the latter, followed by a detailed and formal description in the Methods.

**Fig 1:**
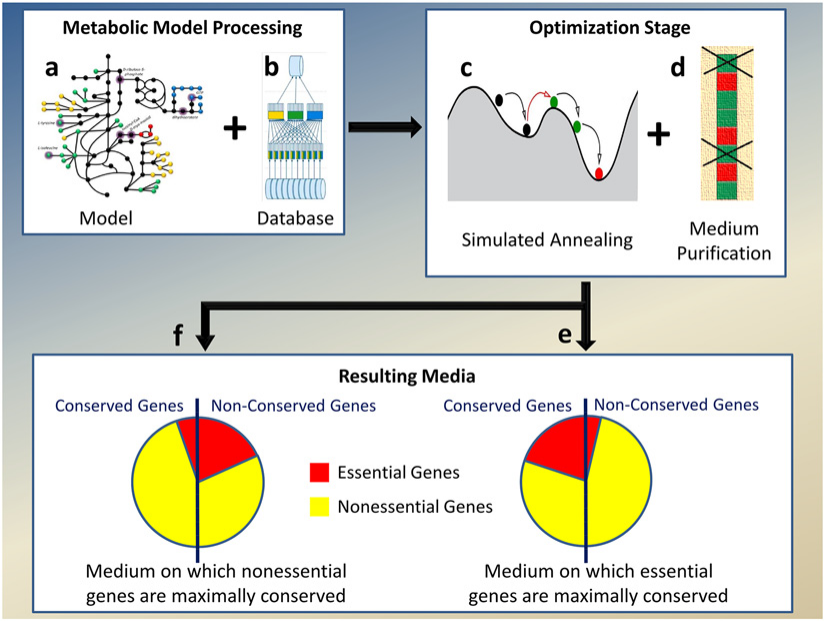
Algorithm for finding a medium with the maximal separation between dN/dS values of essential and nonessential genes. Metabolic models (a) are first preprocessed and databases added to them, to support fast computation of essential genes (b). Simulated Annealing (c) is the first stage of optimization and the resulting medium found is further purified from redundant compounds (d), resulting in the two desired media: those which maximize the differential conservation of essential genes (e) and those which maximize the differential conservation of the nonessential genes (f).

*Media Variation Analysis* (MVA) is a novel generic approach for investigating the behavior of a metabolic model trait, by searching for its minimal and maximal values across all possible media—thus identifying the trait’s feasible range. Applied to the question at hand, Differential Conservation MVA will look for media that maximize and media that minimize the differential conservation of essential genes for a given species. Our algorithm for implementing Differential Conservation MVA is termed *Evolutionary Conservation Of Essentiality Driven Search* (ECOEDS). The algorithm consists of two optimization stages; it receives as inputs a genome scale metabolic model (GSMM), a random starting medium and evolutionary conservation scores for the model genes. ECOEDS outputs a medium, such that the essential and nonessential gene sets identified on it, have the maximal possible separation between their evolutionary conservation scores. ECOEDS searches for such a separation in both directions; once to find media where essential genes are most differentially conserved vs. the nonessential ones, and once, in the inverse direction, finding media where the nonessential genes are maximally conserved vs. the essential ones (Fig 1, Methods 5.1). As part of ECOEDS we have greatly accelerated the running time of existing methods for calculating the set of GSMM essential genes. Among other things, this has enabled us to identify all model genes that are essential under at least one medium (see APE in Methods 5.3 and S1 File 1.2)—a problem for which we are not aware of any current solutions.

*Essential Gene Sets* (EGS) is a media-independent method for partitioning a metabolic model’s genes into essential and nonessential, thus enabling a media-independent analysis showing whether a species tends to follow the KOR-hypothesis or not. The partition is done by defining each gene as essential or nonessential according to its behavior across all possible media (see Methods 5.3 and S1 File 1.2).

## Results

### 3.1 Overview

This large-scale study of evolutionary conservation vs. gene essentiality examines three major research questions: (1) How does the differential conservation of essential genes vary among bacteria? (2) Do bacteria *significantly* differentially conserve their essential genes vs. their non-essential ones? (3) Is the evolutionary conservation of essential genes in bacteria biologically meaningful?

We show that the tendency to differentially conserve essential genes varies strikingly across bacterial species and growth media (Results 3.2 and 3.4), and that some bacteria used in previous experiments are not expected to follow the KOR-hypothesis—possibly explaining the contradictory experimental results obtained in the past (Results 3.2). Overall, we find bacteria tend to follow the KOR hypothesis, albeit weakly. These results were obtained via the use of two new computational approaches—*Media Variation Analysis (MVA)* and *Essential Gene Sets (EGS)*; both methods give a media-independent score (that is, a score determined by surveying all possible *in-silico* growth media) for the differential conservation of an organism’s essential genes.

The third question was tackled by partitioning our bacteria into two groups—those who adhere to the KOR-hypothesis and those who do not. We then looked at a wide range of biological attributes and searched for a significant separation in these attribute values between the two groups. Intriguingly, we did not discover a significant separation of the values in any of the attributes tested (Results 3.5).

### 3.2 Predicting the possible outcomes of KOR Hypothesis experiments

Using Media Variation Analysis we predict the range of possible outcomes across different growth media, for laboratory KOR hypothesis experiments. Across 58 bacteria and the yeast *S*. *cerevisiae* we show that some will always be found to follow the KOR hypotheses, others will never be seen to follow it and some may or may not follow it depending on the growth media. In agreement with previous experimental reports, we predict that *E*. *coli* K12 and *S*. *cerevisiae* (both of which have taken part in contradictory studies) are among the organisms whose tendency to follow the KOR hypothesis is growth-media dependent. We show that across all bacteria used in this study—a significant majority follow the KOR hypothesis.

For most bacteria, the natural environments where they have evolved (and which determined the evolutionary conservation of their genes) are unknown, and the genes essential under these media are hence unknown as well. Though evolutionary conservation estimates can be made, the ‘true’ KOR score, computed according to the genes essential on natural media, remains a mystery. To overcome this obstacle, we perform a Differential Conservation MVA (Methods 5.1)—finding *in-silico* media that maximize and that minimize the differential conservation of essential genes, thus providing upper and lower bounds on the KOR score of a given species. Assuming these bounds can be found, the ‘true’ KOR score is guaranteed to lie within this min-max interval. The intervals defined by the minimal and maximal KOR scores allow classification of organisms into 5 KOR classes (S1 File 2.1): *Strongly KOR (Strongly anti-KOR)* organisms, where the essential (non-essential) genes are differentially conserved across *all possible media*, *Weakly KOR (Weakly anti-KOR)* organisms, where the essential (non-essential) genes are only differentially conserved in some media, and *Undecided* organisms, where neither essential nor nonessential genes were found to be differentially conserved in any media (S1 Dataset sheet “KOR Classification”).

**Fig 2:**
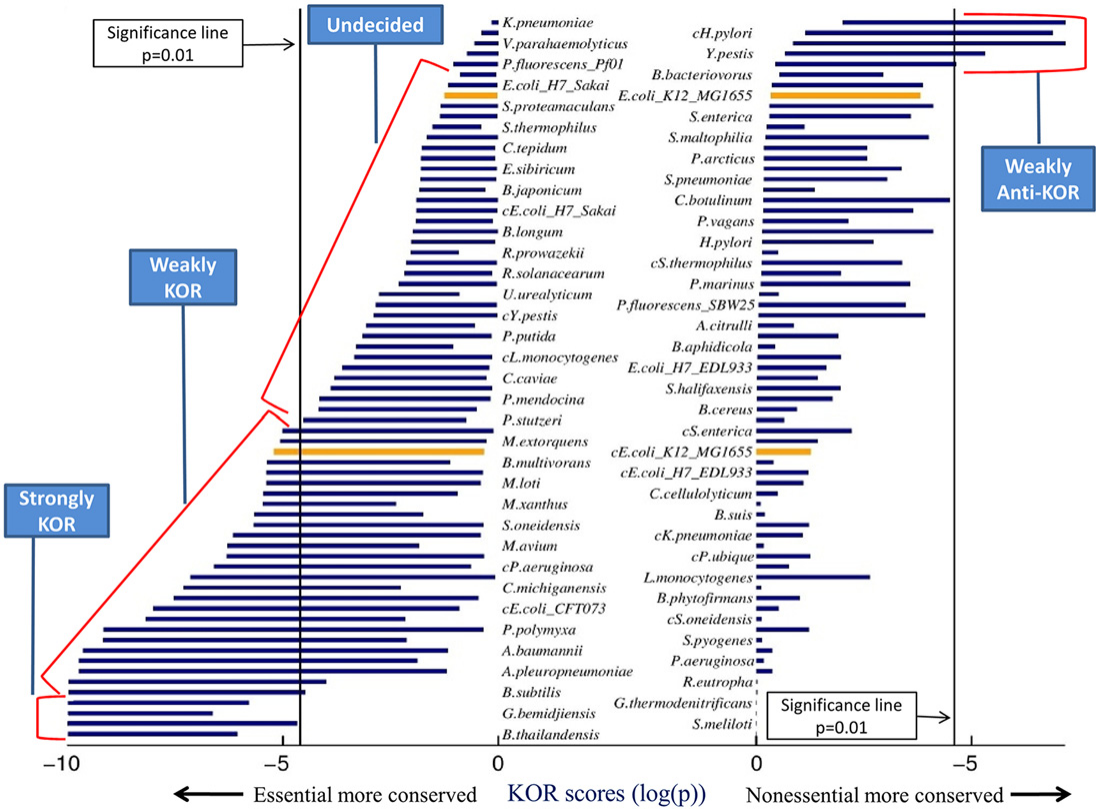
Bacteria model KOR scores across all possible media. Each horizontal bar represents a KOR score interval—the minimum and maximum scores attained by the model across all possible media. Each model is represented by two bars, one on the left and one on the right (bacteria names are presented in two columns for readability only). The left column of bars shows KOR scores testing the hypothesis that the essential genes are differentially conserved, while the right column of bars shows the Anti-KOR scores, that is, testing the hypotheses that non-essential genes are differentially conserved. Models with left bars extending left of the (left) significance line have a medium under which they follow the KOR hypothesis, and analogously, models with right bars extending right of the (right) significance line have a medium under which they follow the anti-KOR hypothesis. Both *E*. *coli* models used in the study are shown in orange (upper one is SEED model). KOR Classes are marked by the blue text boxes. We did not find an organism whose essential genes were differentially conserved in some medium and his nonessential genes were differentially conserved in another medium—this can be seen as no bacterium has both its left and right bars crossing the significance lines.

**Fig 3:**
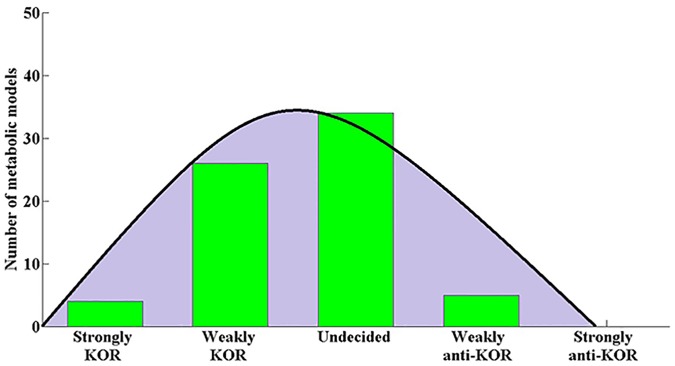
Metabolic model KOR class distribution. The distribution of metabolic models among KOR classes: The (normally-fitted) distribution tends towards the Strongly-KOR class, showing an overall mild tendency of the bacteria studied to conserve the sequence of their essential genes. No bacterial models were found to be Strongly-anti-KOR.

Fig 2 shows the resulting KOR score intervals for the 69 bacteria models surveyed, and Fig 3 shows the distribution of metabolic models among the 5 KOR classes. Four models were found to be strongly KOR, while no models were found to be strongly anti-KOR; fitting a normal distribution to the number of models in the 5 classes shows the mean to lie between Weakly KOR and Undecided, supporting the notion that bacteria tend to follow the KOR hypothesis, albeit weakly. 24 models were classified as Weakly KOR, meaning that these bacteria may comply with the KOR hypothesis according to essential genes found on some lab media, but will not follow the KOR hypotheses when tested on other media. Regarding *E*.*coli K12*, one model was classified as Weakly KOR and the other as Undecided, implying that experimentally determining whether *E*.*coli* follows the KOR Hypothesis or not is media dependent. This may account for several contradicting previous studies which targeted the KOR score of this bacterium [1], [9], [10]. A metabolic model of *S*. *cerevisiae* [27] was analyzed in the same manner and was classified as Weakly KOR, which again may explain previous inconsistent findings with regards to this yeast [4]–[8]. Several bacterial models occupy the Weakly anti-KOR class, meaning that on certain media their nonessential genes are significantly conserved compared to their essential genes—the complete opposite of the KOR hypothesis. We did not find an organism whose essential genes were differentially conserved in some medium and its nonessential genes were differentially conserved in another medium, even though this is theoretically possible.

### 3.3 Validation with gene essentiality experiments

**Fig 4:**
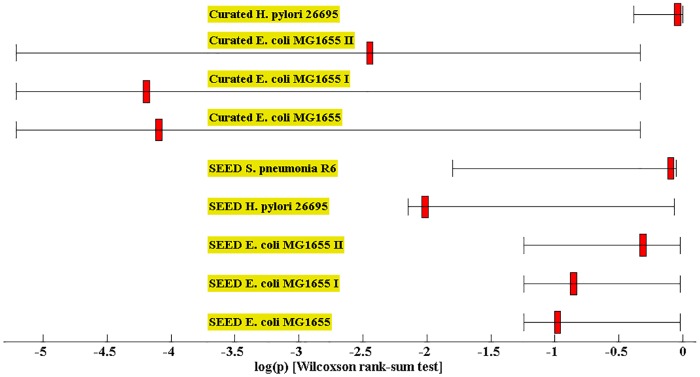
Experimental vs. MVA derived KOR scores. KOR scores were computed for several gene-essentiality datasets from DEG, which were experimentally determined on synthetic lab media. Each horizontal error bar marks the computationally derived KOR score bounds found by ECOEDS and the small red rectangle marks the experimental DEG KOR score. Where available, both SEED and curated models were used. The DEG KOR scores for all organisms (but one—*B*. *thailandensis*, not shown) scored within the predicted computational bounds.

Our Differential Conservation MVA algorithm (ECOEDS) aims to find media that induce maximal and minimal KOR scores. If the algorithm we devised is effective, one would expect the KOR score of any medium and specifically synthetic lab media, to lie within the bounds of these maximal and minimal scores. To evaluate this claim we used the Database of Essential Genes (DEG) to obtain experimentally ascertained sets of essential genes on synthetic lab media [28]. 5 of the bacterial species included in our study have essentiality datasets in DEG, with 3 different datasets for *E*. *coli* K12. Each dataset is composed of a list of bacterium genes with an ‘essential’ or ‘nonessential’ label, and reducing it to include only genes found in the metabolic model results in a partition of the model genes that can then be used to calculate a KOR score. The DEG KOR scores were compared to the maximal and minimal KOR scores found by our algorithm for each bacterium (Fig 4). Across all DEG bacteria (except *B*. *thailandensis*), lab media KOR scores lie within the predicted bounds (Binomial *p* = 0.0029). This agreement serves as an encouraging support for the analysis presented.

### 3.4 Do bacteria follow the KOR hypothesis? An Essential Gene Sets analysis

Using Essential Gene Sets—a partition of genes into essential and nonessential in a manner independent of growth media, we again show that over all bacteria used in this study—a significant majority follow the KOR hypothesis.

Essential Gene Sets (EGSs) is a method to partition genes according to their essentiality behavior across all growth enabling media. Three such distinct sets are defined: (1) genes that are essential across all media (*Always Essential*—*AE)*, genes that are *Never Essential (NE)* and genes that are essential under at least one medium (*All Possible Essential*—*APE*) (Methods 5.3). Obviously, the AE set is contained in the APE set and the NE set is the complement of the APE set. The computation of the AE set for a given metabolic model is performed in a straightforward manner by testing gene essentiality on a rich medium. The computation of the APE set is more challenging and relies on approximation methods (S1 File 1.2).

Given a metabolic model and a division of its genes to the three EGSs, we computed six different KOR scores (AEt, AEr, APEt, APEr, AENEt, AENEr, see below). These were attained from three partitions of the model genes into two distinct sets based on the EGSs, times two statistical tests (Methods 5.3). For example, the AEr and AEt scores were computed by considering the AE set as ‘essential’ and the rest of the genes as ‘nonessential’. The AEr score used a rank-sum test for significance and the AEt score used a t-test. As evident in Table 1, many more bacteria have significantly conserved essential genes than would be attributed to chance. Note that if evolutionary conservation scores were not significantly different among essential and nonessential genes, less than one model on average (0.69 from 69) should exhibit a p-value (KOR score) below 0.01. An analogous analysis calculating anti-KOR scores shows that under most partitions there are no bacteria displaying a differential conservation of nonessential genes (S1 File 1.1). These results are in line with the results obtained from the previous KOR-classes analysis (Results 3.2). Furthermore, KOR-classes and EGS analyses are consistent since all models found to have significantly conserved essential genes under the EGS analysis were classified as Strongly-KOR or Weakly-KOR in the KOR-class analysis (S1 Dataset sheet “EGS KOR Scores”). Both analyses lead to the conclusion that overall, bacteria tend to conserve the sequence of their essential genes more than that of their nonessential genes.

**Table 1:** Bacterial tendency to have differentially conserved essential genes.

KOR score method	Number of models with *p* < 0.01	Binomial p-value
AEt	14	8.90E-15
AEr	6	6.36E-05
APEt	17	3.54E-19
APEr	10	1.88E-09
AENEt	13	2.20E-13
AENEr	7	5.79E-06

The middle column lists the number of bacterial models with a significant KOR score. The right column shows the Binomial p-values that such a number of conserved models will be obtained by chance.

### 3.5 Are KOR scores biologically meaningful?

We show that differential conservation in bacteria is not linked with central biological traits such as growth rate, lifestyle, habitat or phylogenic profiles. We thus call into question the biological relevance of this trait.

The two previous sections showed a considerable variation in KOR scores between different bacteria. To examine whether KOR scores are associated with key biological properties, we partitioned our bacteria into KOR (those who follow the KOR hypothesis) and non-KOR (those who do not follow the KOR-hypothesis) (Methods 5.4) and checked for differences between KOR and non-KOR bacteria across habitat, phylogeny, metabolic and genomic characteristics. Six bacterial attributes were examined: three attributes describing lifestyle and three characterizing bacterial metabolic models and genomes (Methods 5.6). For each attribute, the difference in values between KOR and non-KOR bacteria was tested for significance. Several partitions of the 69 models into KOR and non-KOR groups were tested (Methods 5.4). Interestingly, in all partitions and across all the biological attributes examined, no significant difference between KOR and non-KOR bacteria was found (using a threshold of *p* > 0.01 in a two sided Wilcoxon rank-sum test, and correcting for multiple hypotheses testing). The 5 KOR classes (defined in Results 3.2) provide another more fine-grained partition of the models into KOR and non-KOR groups. Similarly, we looked for a significant difference between the biological attribute values of bacteria from the 5 KOR classes. Using a-parametric ANOVA, yet again no significant separation of values was observed (using a threshold *p* > 0.01 Kruskal-Wallis test).

Looking for a phylogenetic disposition towards the KOR hypothesis, an effort was made to include models from a wide range of bacteria; S1 File Table 2 shows phyla and class data for the models in the study. For each of the methods for splitting bacteria into KOR and non-KOR groups, each phyla and class were checked for enrichment in either KOR or non-KOR bacteria. No such enrichment was found (using a threshold Hypergeometric *p* > 0.01, corrected for multiple hypotheses (S1 File 2.6)).

To assess the potential association between KOR score and bacterial growth habitats, the 58 bacteria in the study were mapped to 77 different environments (S1 Dataset sheet "HG envs") using the GreenGenes databases [29], and to 6 lifestyles (S1 Dataset sheet "HG envs") using the 6-class database [30]. For each of the methods for partitioning bacteria into KOR and non-KOR groups, each environment and lifestyle were checked for enrichment in either KOR or non-KOR bacteria. No significant enrichment was found in any of the cases (using a threshold Hypergeometric *p* > 0.01 corrected for multiple hypotheses (S1 File 2.6)).

## Discussion

Using metabolic model analysis across all *in-silico* media we show that current laboratory experiments aimed at determining whether a certain bacterium follows the KOR hypothesis or not, are prone to bias induced by the laboratory growth medium used. We bring evidence that these experiments may have strikingly different results on different growth media and we present this as a possible explanation to decades of contradictory studies [1], [9], [10], [4]–[8]. We show that overall, bacteria *do* have a tendency to follow the KOR hypothesis, but that this tendency does not seem to be biologically meaningful since bacteria that follow the hypothesis and bacteria that do not, could not be differentiated according to central biological traits.

The analyses presented here investigate the evolutionary conservation of essential genes in a media-independent manner, that is, across all feasible *in-silico* growth-enabling media. We thus circumvent the bias induced by determining gene essentiality on synthetic lab media that do not necessarily reflect the natural habitats of the species. We present two new cross-media analyses: (1) MVA, which estimates the range of differential evolutionary conservation across all media and (2) Essential Gene Sets, which splits the genome into essential and nonessential genes, according to their essentiality behavior over all possible media. Using both methods we show that there are more cases of bacteria conserving the sequence of their essential genes vs. their nonessential genes than would be attributed to chance. We further demonstrate that bacteria lie on a wide spectrum of evolutionary conservation of essential genes, ranging from those who differentially conserve the sequence of their essential genes to those who differentially conserve the sequence of their nonessential genes. Therefore, when testing the KOR hypothesis on different bacteria or even on the same bacterium in different media—disparate results may be obtained and contradictory conclusions drawn. Partitioning bacteria into KOR and non-KOR groups, as well as according to the finer grained KOR-classes, no tested biological attribute, including bacterial habitats and phylogenetic origins, distinctly characterizes any of the groups or classes. Thus, the functional significance of the KOR hypothesis, and the biological origin of the variance in evolutionary conservation of essential and nonessential genes remain unclear.

We present MVA—a generic approach that may be useful when studying the variance of other metabolically-related traits across all media. Differential Conservation MVA was made possible by new methods for fast calculations of essential genes in metabolic models (S1 File 2.2). In fact, any metabolic model objective function that involves essential genes may be sped up using these methods.

This study has a few caveats that should be mentioned: First, it is a computational study, based on computational predictions that present only a rough approximation of the underlying reality. Second, as this study is based on genome-scale metabolic models, only metabolic genes were taken into account, and the overall picture may change when considering full genomes. Third, conclusions pertaining to the bacterial domain were voiced based on the 58 bacterial species studied, although these do not necessarily constitute a representative sample of all bacteria (if such a sample exists). Fourth, 56 of the 69 models used were automatically generated by the Model Seed algorithm [26], mostly having a lower level of accuracy in their prediction of essential genes compared to human curated models [26]. However, our key findings remained similar when the analyses were reduced to the 13 human curated models in our collections (S1 File 1.7).

In summary, this paper presents two novel computational approaches for evaluating gene essentiality in a media-independent manner. Furthermore, the array of bacteria and media tested is far larger than any previous study dealing with evolutionary conservation of essential genes. It brings new insights to an age old question concerning the differential evolutionary conservation of essential genes and, for the first time, describes a multi-species cross-media analysis that may explain the inconsistent results reported in past studies.

## Methods

### 5.1 Media Variation Analysis (MVA)

Media variation analysis (MVA) is a novel generic approach for investigating the behavior of a GSMM trait, by searching for the minimal and maximal values of the trait across all possible media, thus identifying its feasible range. For example, the MVA of growth-rate for a certain GSMM would look for the maximal and minimal growth rates across all possible media. In this case, the answer is trivial since the minimal growth-rate is zero and the maximal growth-rate will be attained on a rich medium. For other GSMM traits however, discovering the possible range of values may be computationally difficult due to the exponentially large space of *in-silico* media. In this study we present a two-stage optimization algorithm to implement MVA. We then use this algorithm to compute the cross-media minimal and maximal values of two basic traits of interest: (1) the differential conservation of essential genes and (2) the number of essential genes.

### 5.2 Differential Conservation MVA algorithm (ECOEDS)

Differential Conservation MVA requires finding media with maximal/minimal differential conservation of essential genes. This was implemented by a two stage optimization algorithm that receives as inputs a GSMM, a random starting medium and evolutionary conservation scores for the model genes. The algorithm outputs a medium, such that the essential and nonessential gene sets it defines have the maximal possible separation of evolutionary conservation scores. The first stage of the algorithm uses Simulated Annealing to search the media space and once it converges to a medium that maximizes the objective stated above, a second Purification stage filters redundant intake (exchange) reactions.

To perform Differential Conservation MVA, two variants of ECOEDS were run on each model, one with the objective of maximizing the conservation of the essential vs. the nonessential genes and one with the objective of maximizing the conservation of the nonessential vs. the essential genes. In the following paragraphs we refer to the variant which aims to maximize the differential conservation of essential genes (the workings of the opposite variant are analogous).

#### 5.2.1 The Simulated Annealing stage

The Simulated Annealing search is conducted over the GSMM metabolite intakes (exchange reactions) space. To simplify the search, media were reduced to a binary version; each intake reaction is either opened or closed (can/cannot carry flux). To converge to an optimal solution, Simulated Annealing requires a scoring function that maps each point to a real number. For a given search point (medium), the essential and nonessential genes are determined from the model and the dN/dS estimates’ split is computed accordingly. The resulting score for that point is log(*p*) of the one-sided Wilcoxon rank-sum p-value, which denotes the significance of the differentiation in conservation scores (Assuming the median of the essential set is lower than the median of the nonessential set. The reverse is assumed to obtain the opposite objective—maximize the differential conservation of the nonessential genes). The Simulated Annealing search proceeds for a specified number of iterations and then returns the best medium (lowest log(*p*)) encountered (S1 File 2.3)).

#### 5.2.2 Purification Stage

A medium ***M*** resulting from the Simulated Annealing stage is a list of all the model exchange reactions, some open and some closed. The Purification stage filters redundant open exchange reactions in ***M***, by randomly choosing an open intake and closing it if viability is kept and the KOR score (for the new medium) is at least as good as ***M***’s (S1 File 2.3). This random removal is repeated until no open exchange reactions can be closed while keeping with the previous constraints.

#### 5.2.3 Performance

Several tests assessing the performance of the Differential Conservation MVA optimization algorithm show that it most likely converges to the optimal or near optimal solution for most models. 250 searches (each involving choosing a random media starting point and performing simulated annealing and purification steps described above) were performed for each model to obtain the results presented, and increasing the number of searches failed to yield media with significantly better KOR scores. Furthermore, the algorithm finds media which consistently score better than random and synthetic media (see Results 3.3 and S1 File 2.4).

### 5.3 Essential Gene Sets—AE, PE, APE and NE genes

In order to classify genes as essential or nonessential in a media-independent manner, we defined the notion of *Essential gene sets*: Given a metabolic model, genes that are found to be essential under a rich medium (all metabolite intake reactions are open) will be essential under any medium, since any medium is a proper subset of the rich medium. We call these *Always Essential* (AE) genes. We term *All Possible Essential* (APE) genes, those genes for which there exists a medium (even if only one) under which they are essential. Genes that are not in APE are termed *Never Essential* (NE), and APE genes that are not AE are termed *Potentially Essential* (PE). Clearly

|APE|+|NE|=|AE|+|PE|+|NE|=|model genes|

AE⊆APE,AE∪PE=APE

$$APE=\underset{m\in \text{in-silico-media}}{\cup }essential(m)$$

Where *essential*(*m*) is the set of genes essential on a medium *m*.

Comprehensively computing the APE set is hard, but a close approximation was made (S1 File 1.2). The approximation relies on a novel and fast method for computing gene essentiality that enabled us to: (a) construct the APE set by gathering all genes found to be essential in a large set of randomly sampled growth-enabling media, and (b) verify that the APE set is complete with high likelihood, by sampling an additional set of thousands of random growth-enabling media, and verifying that no new essential genes can be found.

### 5.4 KOR score and KOR vs. non-KOR partitions

The **KOR score** is a statistical measure of the differential separation of evolutionary conservation estimates between essential and nonessential genes. Given evolutionary conservation estimates and a partition of the genes into ‘essential’ and ‘nonessential’, a KOR score can be computed. To obtain media-independent KOR scores, three media-independent ways to partition the genome into essential and nonessential based on Essential Gene Sets were used, as summarized in Table 2.

**Table 2:** Summary of metabolic genome partitions into essential and nonessential genes according to Essential Gene Sets.

Partition Name	‘essential’ set	‘nonessential’ set
AE-partition	AE genes	All-but-AE genes
APE-partition	APE genes	NE genes
AENE-partition	AE genes	NE genes

Three methods are presented for partitioning the genome into essential and nonessential gene according to Essential Gene Sets:

AE-partition is equivalent to the partition done in previous related experimental research, had the experimental essential gene set been determined on a rich medium.

APE-partition is similar to the partition done in previous related experimental research, had the experimental essential gene set been determined on a poor medium.

AENE-partition produces a marked separation, possibly helping overcome metabolic model inaccuracy. AE is the core group of essential genes with a higher probability of aligning with essential genes from experimental data. Similarly, NE are more likely to overlap with nonessential experimental genes. This partition does not cover all metabolic genes, leaving out the PE set.

Once a partition is defined, dN/dS estimates of the two groups are compared using two statistical tests to check for significance:

1. One-sided Wilcoxon rank-sum test.
2. Single tailed t-test for log dN/dS, assuming bacterial dN/dS approximately follow a log-normal distribution [31].

To assure that a significant KOR score implies a significant tendency towards the KOR Hypothesis, both statistical tests are one-sided, assuming the median/mean value of the ‘**essential’** group is lower. An analysis of Essential Gene Sets anti-KOR scores, assuming that the median/mean value of the ‘**nonessential’** group is lower can be found in S1 File 1.1 (S1 Dataset sheet "EGS KOR scores"). The three partitions and two statistical tests combine to produce six methods for assigning KOR scores in the EGS approach, as summarized in Table 3. Reassuringly, KOR scores for the same model obtained via these 6 definitions are highly correlated (average Spearman's *ρ* = 0.707,*p* < 0.0001). Note that a different KOR score may be obtained for two (different) models of the same bacterium even using the same KOR score method, as the two models may include different genes and may yield different essentiality predictions.

**Table 3:** Summary of methods for assigning KOR score with EGS partitions.

KOR score method	Description	KOR score method	Description
AEr	AE-partition with rank-sum test	AEt	AE-partition with t-test
APEr	APE-partition with rank-sum test	APEt	APE-partition with t-test
AENEr	AENE-partition with rank-sum test	AENEt	AENE-partition with t-test

Three ways to partition the genome along with two different statistical tests for significance lead to six methods for assigning a KOR score to a metabolic model genome.

Each method for assigning a KOR score allows us to rank the 69 models from smallest to largest KOR score, for a total of 6 different rankings. For each ranking, a threshold is used to separate the models into two groups—KOR and non-KOR. We tested 6 different thresholds (10, 20, 30, 40, 50 and 60), each threshold determining the number of models in the KOR group. For example, for threshold = 20, the 20 models with the best KOR score (lowest p-value) were put in the KOR group and the rest in the non-KOR group. The 6 rankings and 6 thresholds result in 36 ways to partition the models into KOR and non-KOR bacteria, which were used in the analysis of biological attributes.

### 5.5 dN/dS Estimates

The basic measure of the selection pressure acting on protein-coding sequences is the ratio of the number of nonsynonymous substitutions per nonsynonymous site to the number of synonymous substitutions per synonymous site, also known as dN/dS (or Ka/Ks). Substitutions are determined by comparing the genome in question with the genome of a closely related reference species or strain. Genes under purifying selection will display a lower dN/dS whereas positive selection, which increases dN, will result in increased dN/dS values [31].

dN/dS estimates for some 400 bacteria were obtained from an updated version of the Alignable Tight Genomic Clusters (ATGC) database (unpublished work; [32]). Bacteria for the analysis were chosen from a set of 139 ATGC groups with the aim of having just one pair (bacterium and reference bacterium) per group. Pairs were chosen so that the genome-wide synonymous substitution rate would be in the range of 0.25–1.5, preferentially choosing pairs such that the median dS (among all genes shared between the two genomes) will be as close to 0.75 as possible. The upper limit was chosen to keep dS below saturation (i.e., keep it a reliable estimate) for most of the genes in the genome, and the lower limit to reduce the fraction of proteins in the genome that have a very low nonsynonymous substitution rate (which are again unreliable) [32].

### 5.6 Biological attributes

For a full description of the biological attributes used in this study, scores and references—see S2 Dataset sheet "Biological Attributes".

## Supporting Information

S1 Dataset Results and Sources. An Excel sheet containing information about the data used in this study and the results obtained. See detailed description of contents in section 3 of S1 File.
(XLSX)

S2 Dataset Results and Sources. An Excel sheet containing information about the data used in this study and the results obtained. See detailed description of contents in section 3 of S1 File.
(XLSX)

S1 File Supplementary results, methods and information. Supplementary results include further analysis of the Essential Gene Sets and of the relation between the KOR score and biological attributes. Supplementary methods include a detailed description of how the KOR classes were obtained, a full description of the MVA algorithm, the processing done on the metabolic models in this study and some remarks regarding multiple hypotheses testing. S1 File includes a detailed description of S1 Dataset and S2 Dataset.
(DOCX)
