# Supplementary material for: Evolutionary Conservation of Bacterial Essential Metabolic Genes across All Bacterial Culture Media
Source: PLoS One. 2015 Apr 20;10(4):e0123785. doi: 10.1371/journal.pone.0123785 (PMC4403854; doi:10.1371/journal.pone.0123785)
Supplement: S1 File — Supplementary results include further analysis of the Essential Gene Sets and of the relation between the KOR score and biological attributes. Supplementary methods include a detailed description of how the KOR classes were obtained, a full description of the MVA algorithm, the processing done on the metabolic models in this study and some remarks regarding multiple hypotheses testing. S1 File includes a detailed description of S1 Dataset and S2 Dataset. (DOCX) [file pone.0123785.s003.docx]

Supplementary Results, Methods and Information

1 Supplementary Results 2

1.1 Media-independent KOR scores computed via an Essential Gene Sets analysis 2

1.2 Essential Gene Sets – approximating the APE 3

1.3 Essential Gene Set sizes 4

1.4 KOR scores and Biological Attributes 4

1.5 KOR scores and Phylogeny 5

1.6 KOR scores and Habitat 6

1.7 Curated-only model analysis 6

2 Supplementary Methods 7

2.1 KOR classes from Differential Conservation MVA 7

2.2 Gene essentiality fast calculations 6

2.3 ECOEDS SA and Purification details 7

2.4 ECOEDS efficacy and convergence 8

2.5 Metabolic-model processing 11

2.6 Correcting for multiple hypotheses 15

3 Supplementary Information 16

4 Supplementary Section Bibliography 17

# Supplementary Results

## Media-independent KOR scores computed via an Essential Gene Sets analysis

As explained in Methods 5.4, the KOR scores for the Biological analysis were calculated with a ‘left’ tail t-test and rank-sum test, that is, assuming the mean/median of conservation values for the ‘essential’ set is lower. To complete the analysis, the same partitions with the ‘right’ version of the statistical tests were run, to obtain anti-KOR scores (assuming the mean/median of conservation values for the ‘nonessential’ set is lower). Table 1 presents both analyses. While there are more significant KOR Scoring models than would be expected by chance, the same is not true for anti-KOR Scoring models. This asymmetry strengthens the conclusion that bacteria tend to differentially conserve the sequence of their essential genes relative to their nonessential genes. All EGS KOR and anti-KOR scores are found in S1 sheet “EGS KOR scores”.

Table 1 – Number of significant KOR Scoring bacteria

| **KOR score Method** | **Number of models with**  $\boldsymbol{p<0.01}$ | **Binomial p-value** |
| --- | --- | --- |
| Left Tail: KOR score – assuming essential genes are more conserved | | |
| AEt | 14 | 8.90E-15 |
| AEr | 6 | 6.36E-05 |
| APEt | 17 | 3.54E-19 |
| APEr | 10 | 1.88E-09 |
| AENEt | 13 | 2.20E-13 |
| AENEr | 7 | 5.79E-06 |
| Right Tail: anti-KOR score – assuming nonessential genes are more conserved | | |
| AEt | 0 | 0.4998 |
| AEr | 2 | 0.1196 |
| APEt | 0 | 0.4998 |
| APEr | 3 | 0.0270 |
| AENEt | 0 | 0.4998 |
| AENEr | 0 | 0.4998 |

The number of bacterial models showing a significant KOR and anti-KOR score are listed in the middle column, across the six media-independent methods for assigning a KOR score. The right column shows the Binomial p-values that such a number of conserved models will be obtained by chance.

## Essential Gene Sets – approximating the APE

Comprehensively computing the APE set is difficult, but an efficient approximation was made. To approximate the APE set, the essential genes for several media types were calculated. Any gene found to be essential on any one of the media was included in the APE set. The media used for the approximation:

1. Media output from 250 ECOEDS runs
2. A large set of random media (thousands of instances per model).
3. Media from an ECOEDS variant whose objective function was to find a medium with the maximal number of essential genes.

The APE gene set for a certain model is complete, if it accounts for all the genes which could possibly be essential under any medium. The APE set can be said to be 'sufficiently complete', if encountering a medium which adds a new gene to the set is a rare event. To test this, for each model a large set (tens of thousands) of random media were computed (not the random media used to approximate the APE) and their essential genes calculated. No medium was found to add a new gene to the set (for each model) and so the APE sets in use were considered sufficiently complete. The APE can be exactly calculated over any finite media (1, 2 above).

## Essential Gene Set sizes

Essential Gene Set sizes across all models are shown in Fig1 (for Figure data see S1 sheet "Essential Gene Sets").

Fig1 – Size of Essential Gene Sets


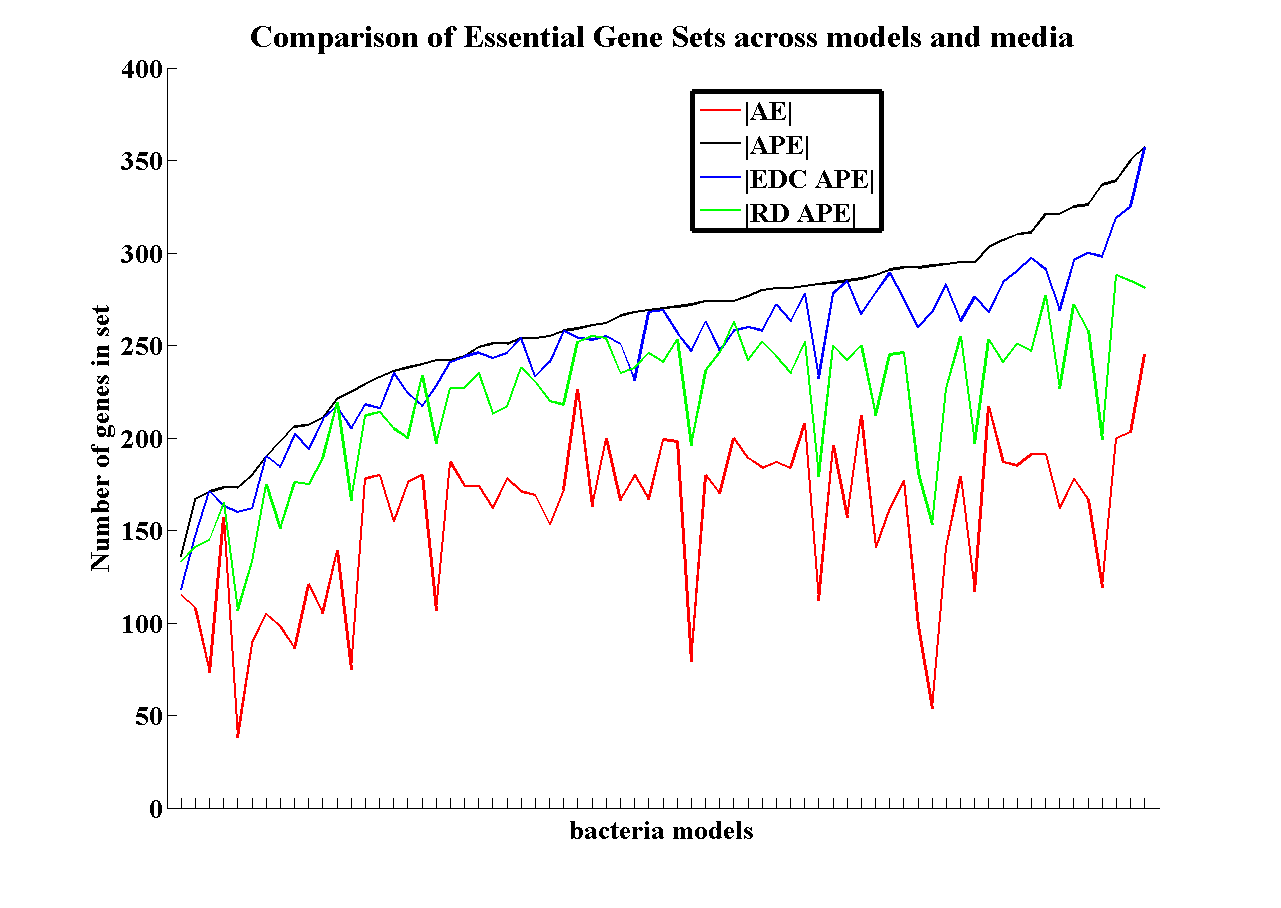


The sizes of AE and APE gene sets are compared between all models on two different media: (1) 250 randomly generated media (RD) and (2) 250 ECOEDS results (EDC). The AE and APE sets are not media dependent. RD APE is the APE set approximated on the set of random media and EDC APE is the APE set approximated on EDC media. The plot is arranged to display species sorted by their $\text{|APE|}$.

## KOR scores and Biological Attributes

We used three biological attributes which classify bacteria lifestyle:

1. Data mapping bacteria into 153 environments ("greengenes") from Chaffron et al.[1]. Each bacterium receives a score consisting of the number of environments it was mapped to.
2. Data classifying bacteria into one of 6 lifestyles ("6-class") according to the multiplicity of environments it inhabits, was taken from Parter et al.[2]. Each bacterium received a score from 1 to 6, 1 being obligate and 6 being terrestrial (highly versatile) bacteria.
3. Data containing bacterial samples from several locations on the human body ("HMP") was taken from the Human Microbiome Project[3].

We used three attributes that characterize bacterial metabolic models and genome:

1. Genome size – from the ATGC database[4]
2. Model Complexity – defined as the number of non-zero elements in the metabolic model stoichiometric matrix.
3. |AE| – the size of the Always Essential gene group, computed on all metabolic models.

For more information on the attributes see (S2 sheet “Biological Attributes”).

For each attribute, the values for KOR and non-KOR bacteria were tested for a significant difference. Checking with 36 KOR vs. non-KOR partitions over the 6 attributes, no significant difference between the two groups' values was found ($p>0.0$1 two sided Wilcoxon rank-sum test).

## KOR scores and Phylogeny

Looking for a phylogenetic disposition towards the KOR hypothesis, an effort was made to include models from a wide range of bacteria; Table 2 shows phyla and class data for the models in the study.

Table 2 – Phylogeny of bacteria in study

| Lineage Rank | Name | # of models | Lineage Rank | Name | # of models |
| --- | --- | --- | --- | --- | --- |
| Phylum | *Actinobacteria* | 3 | Class | *Actinobacteria* | 3 |
|  | *Clamydiae* | 1 |  | *Alphaproteobacteria* | 7 |
|  | *Clorobi* | 1 |  | *Bacilli* | 11 |
|  | *Cyanobacteria* | 1 |  | *Betaproteobacteria* | 6 |
|  | *Firmicutes* | 13 |  | *Chlorobia* | 1 |
|  | *Proteobacteria* | 49 |  | *Clamydiia* | 1 |
|  | *Tenericutes* | 1 |  | *Clostridia* | 2 |
|  |  |  |  | *Gammaproteobacteria* | 36 |
|  |  |  |  | *Mollicutes* | 1 |
|  |  |  |  | *Prochlorales* | 1 |

The phyla and classes of all bacteria in the study are listed with the number of bacteria in each taxonomic group.

For each of the methods for partitioning bacteria into KOR and non-KOR, each phyla and class was checked for enrichment in either KOR or non-KOR bacteria. No such enrichment was found (Hypergeometric $p>0.0$1 corrected for multiple hypotheses (S3 ‎2.6)) apart from one exception: For the AEr partition with threshold=50, class *Gammaproteobacteria* was found to be enriched in non-KOR bacteria (Hypergeometric $p=1.05\times{10}^{-4}$). After correction for multiple hypotheses, the threshold for significance in this test was ${{0.01}/{10}}/3=3.33\times{10}^{-4}$, just slightly higher than the Hypergeometric enrichment score and so we deem it not significant.

## KOR scores and Habitat

To assess the connection between habitat and KOR bacteria, the 58 bacteria in the study were mapped to 77 different environments (S1 sheet "HG envs") using the GreenGenes databases [1]. For each of the 36 KOR vs. non-KOR partitions, each environment was checked for enrichment in either KOR or non-KOR bacteria. To reduce noise, only environments with more than a certain number of applicable models (values of 10, 15, and 20 were tested) were used in the analysis. No enrichment was found (Hypergeometric $p>0.0$1 corrected for multiple hypotheses (S3 ‎2.6)).

Similarly, the bacteria were mapped to 6 lifestyles (S1 sheet "HG envs") by the 6-class database[2]. As with the habitat analysis, none of the lifestyles were found to be enriched in either KOR bacteria, non-KOR bacteria, or in bacteria from the 5 KOR classes.

## Curated-only model analysis

Repeating all KOR score and biological attribute analysis with human-curated models alone, did not change the result and no significant difference between the biological attribute values of the two groups was observed ($p>0.0$1 using a two sided Wilcoxon rank-sum test). Similarly, restricting the phylogeny and habitat enrichment analysis to human-curated models did not change the previous results.

# Supplementary Methods

## KOR classes from Differential Conservation MVA

Differential Conservation MVA aims to find the media that maximize and that minimize the differential conservation of essential genes for a given metabolic model. To perform this analysis on a given model, two variants of ECOEDS were used:

1. ECOEDS: Maximize conservation of essential vs. the nonessential genes. This was run 250 times resulting in 250 media. Each medium was given a score signifying the statistical separation between dN/dS values of essential and nonessential genes. The score was the one-sided Wilcoxon rank-sum p-value, assuming the median value for the essential set is lower than the nonessential set (referred to as ‘left tail Wilcoxon’). We will refer to the medium with the lowest p-value (of the 250) as M1.
2. Anti-ECOEDS: Maximize conservation of nonessential vs. essential genes. This was run 250 times resulting in 250 media. Each medium was given a score signifying the statistical separation between dN/dS values of essential and nonessential genes. The score was the one-sided Wilcoxon ranksum p-value, assuming the median value for the nonessential set is lower than the essential set (referred to as ‘right tail Wilcoxon’). We will refer to the medium with the lowest p-value (of the 250) as M2.

After running ECOEDS and anti-ECOEDS on the 69 models in the study we were left with 69 M1 and 69 M2 media. For each of the 138 media we calculated the essential genes it defines and then ran a ‘left tail Wilcoxon’ and ‘right tail Wilcoxon’ on it. We labeled as "KOR" those media (M1 or M2) who showed a significant result with the ‘left tail Wilcoxon’, "anti-KOR" those media who showed a significant result with the ‘right tail Wilcoxon’ and the rest were labeled as "Undecided". We then classified all bacteria models into **5 *KOR classes*** according to their M1 and M2 labels.

Table 3 details the KOR classes, Fig2 depicts the classification scheme and Fig3 depicts a comparison between KOR classes and the KOR vs. non-KOR partition, a partition used in the analysis of the biological attributes. All model KOR classes are found in S1 sheet "KOR Classification".

Table 3 – KOR classification results

| Terminology | | | | |
| --- | --- | --- | --- | --- |
| KOR Class Name | Definition | | | |
| **Strongly KOR** | KOR in both ECOEDS and anti-ECOEDS runs. Even when median dN/dS of nonessential genes is minimized and median dN/dS of essential genes maximized – essential genes are still significantly more conserved. | | | |
| **Weakly KOR** | KOR in ECOEDS and Undecided in anti-ECOEDS. Definition of species as KOR or non-KOR is media dependent | | | |
| **Undecided** | Undecided in both ECOEDS and anti-ECOEDS. Across all media, neither essential nor nonessential genes were significantly more conserved. | | | |
| **Weakly**  **anti-KOR** | Undecided in ECOEDS and anti-KOR in anti-ECOEDS. | | | |
| **Strongly**  **anti-KOR** | Anti-KOR in both ECOEDS and anti-ECOEDS. No model was found to occupy this class | | | |
| Results | | | | |
| KOR classification | **Strongly KOR** | **Weakly**  **KOR** | **Undecided** | **Weakly**  **anti-KOR** |
| Number of models | 4 | 26 | 34 | 5 |
| Representative Models | *B.thailandensis*  *S.meliloti* | *L.monocytogenes*  c*P.aeruginosa*  *cE.coli_K12_MG1655* | *B.aphidicola*  c*P.ubique*  *E.coli_K12_MG1655* | *K.pneumoniae*  *Y.pestis*  c*H.pylori* |

Table holds the definitions of the KOR classes and the number of metabolic models found to reside in each class. A 'c' before the bacterium name (e.g. "cS.oneidensis") signifies a manually curated metabolic model.


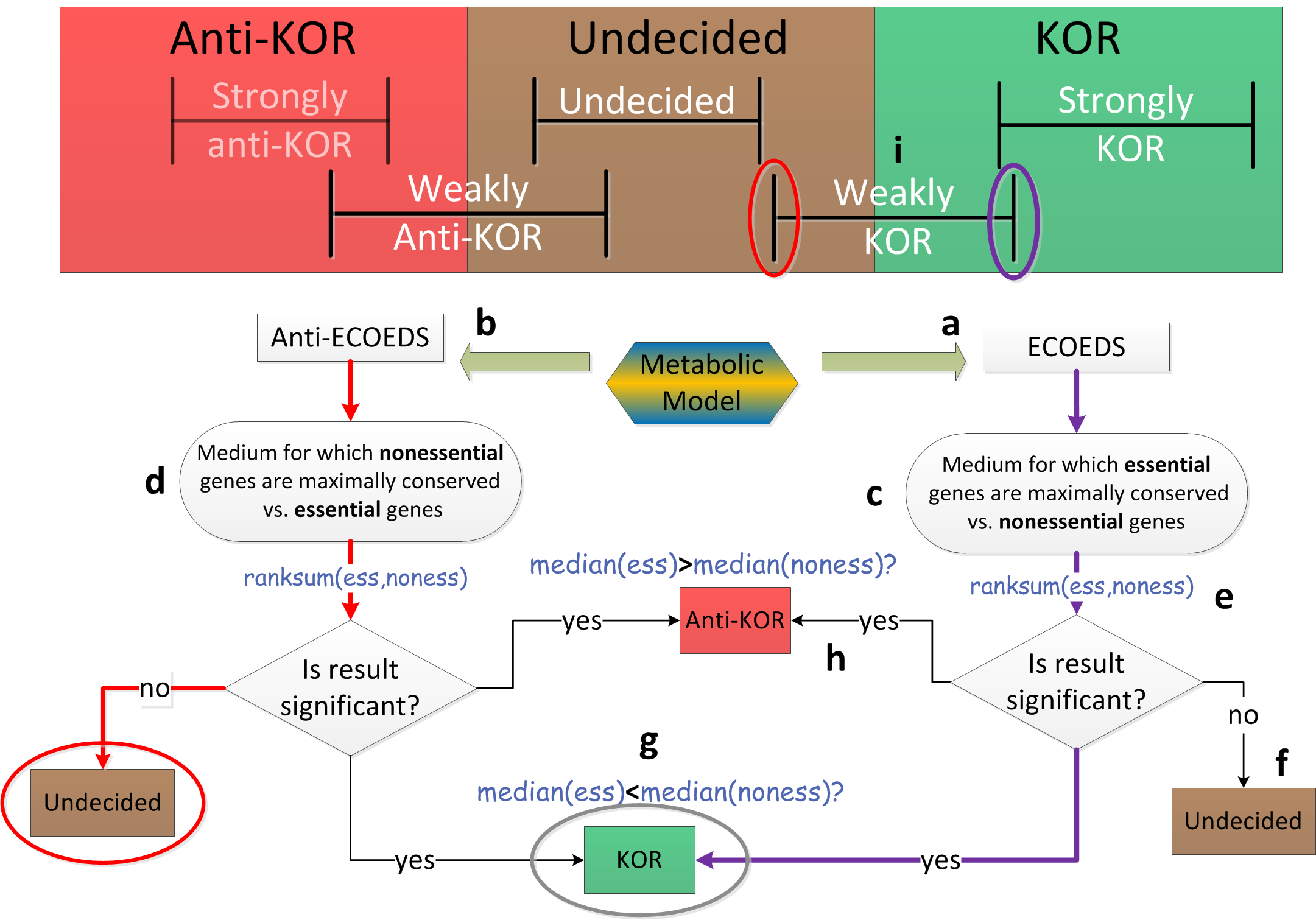


Supp. Table 4 – KOR classification scheme

Fig2 – KOR classification scheme

Each metabolic model is processed twice, once with ECOEDS (a) and once with anti-ECOEDS (b). Both runs result in a medium maximizing the differential conservation between essential and nonessential genes (c,d). After essential genes are computed on the medium, a Wilcoxon rank-sum test is run on the dN/dS of the essential and nonessential gene groups (e) resulting in a classification: if the result is not significant – the run in question is classified Undecided (f), if significant with essential genes more conserved – KOR (g) and if significant with nonessential genes more conserved – anti-KOR (h). The final classification into the 5 KOR classes depends on the combined results from both runs (i).

Fig3– KOR classes and KOR/non-KOR partition comparison


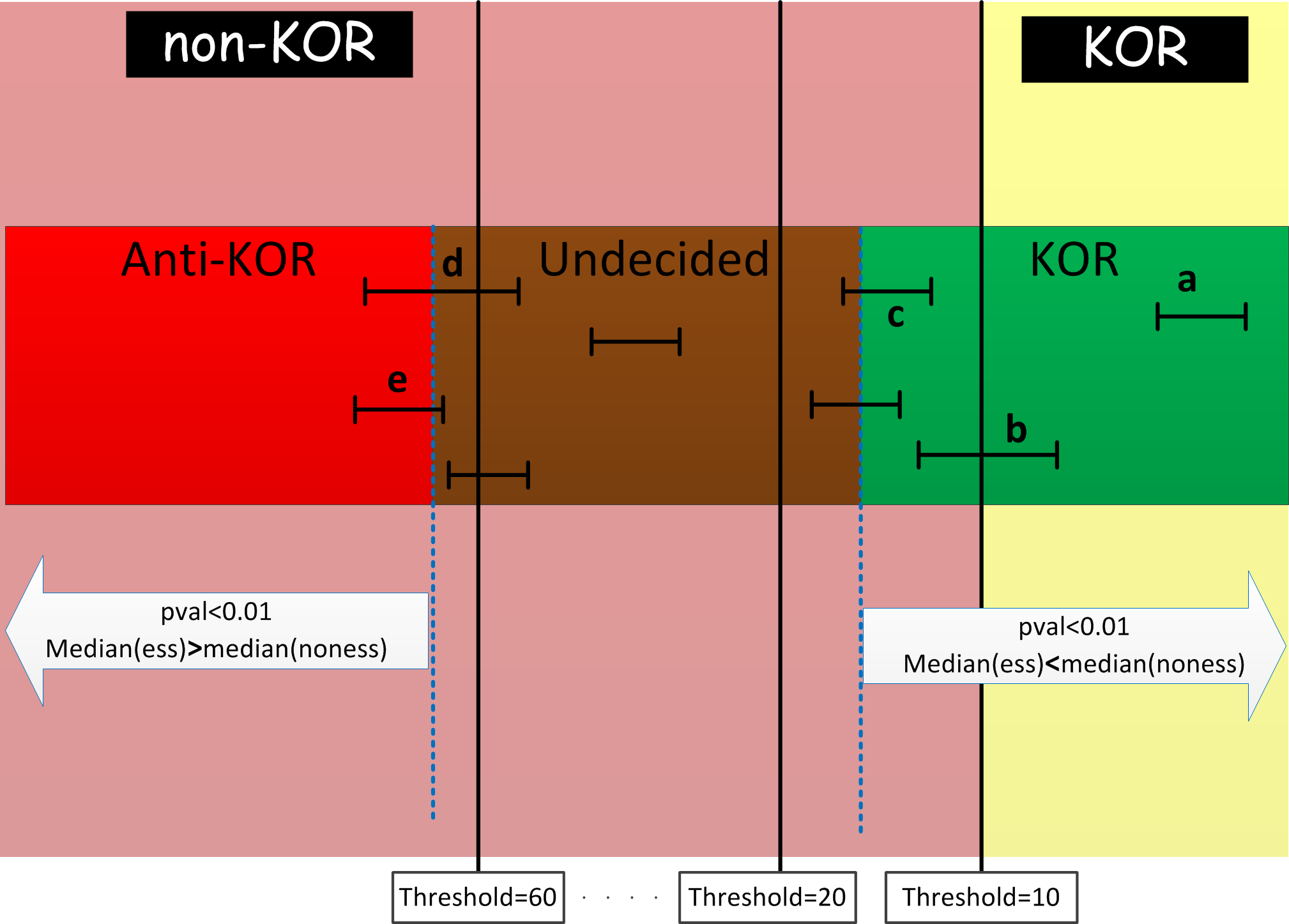


Each horizontal bar represents a model with its M1 and M2 labels – KOR, Undecided and anti-KOR written in black. The KOR vs. non-KOR split depicted with white on black background is threshold dependent. Several thresholds are shown and background is split according to **Threshold=10**. **a** and **b** are classified as Strongly-KOR and will be included in the KOR side whereas **c**, classified as Weakly-KOR, will not. However, for **Threshold=20**, **a**,**b** and **c**, although the KOR classification did not change, will all be in the KOR group. Threshold locations are approximate.

## Gene essentiality fast calculations

A gene is considered essential (*in silico*), if constraining zero flux through all the reactions for which it is a necessary catalyst and solving for maximum biomass, results in negligible biomass production. To find the essential genome, this process of gene knockout is repeated over all genes. Since only viability and not growth rate is important, essentiality calculations are fairly robust to the definition of cellular objective function. Using the biomass objective, the list of essential genes was found to be robust to minimal growth threshold (${10}^{-15}<\text{threshold}<{10}^{-7}$).

In both the ECOEDS SA and Purification stages, calculating a KOR score for each medium necessitates the calculation of the essential genes on that medium. Running 250 SA searches and purifying the 250 results over 69 models required more than 8M genome essentiality calculations. In order to speed these up, several aspects of the model were utilized and some optimization methods used:

### Static preprocessing per model

1. A gene is an essential catalyst of a reaction if it, or a complex of which it is a part, is the only one catalyzing the reaction. Accordingly, each model gene $g$ was given a list $Cat\left( g \right)$ of reactions for which it is an essential catalyst:

$Cat\left( g \right)=\{r_{j_{1}},r_{j_{2}},\ldots,r_{j_{n}}\}$.

1. Some genes have the same $Cat\left( g \right)$; therefore only unique elements of $Cat$ were used in the calculations, and the results were later mapped to all genes.
2. Genes with an empty $Cat$ list are never essential.
3. AE genes were computed (by calculating the essential genes on a rich medium) and stored. All $g\in\text{AE}$ are automatically essential and do not need to be tested.
4. A set of reactions $R$ is calculated, see Dynamic processing per medium
5. The APE set was approximated (‎1.2) and stored. For a given medium, only $g\in\text{APE}$ needs to be tested for essentiality.

### Dynamic processing per medium

1. $R=\{r_{j_{1}},r_{j_{2}},\ldots,r_{j_{n}}\}$ is said to *cover* a set of genes $G=\{g_{i_{1}},g_{i_{2}},\ldots,g_{i_{m}}\}$ if

$$\forall g\in G Cat\left( g \right)\subseteq R$$

A reaction set $R$ can be found such that $R$ is small compared to the full set of reactions in the model, but covers a large portion of the genome.

1. An iMAT like Mixed Integer Linear Programming optimization [5] is solved, looking for a network reaction flux mapping such that the biomass is non-zero and as many reactions in $R$ carry zero flux.
2. If the MILP solution is successful, a set of reactions $T\subseteq R$ is known to be able to carry zero flux when the biomass is non-zero. Genes covered by $T$ are therefore not essential on this medium.

## ECOEDS SA and Purification details

ECOEDS was run on Matlab R2013b using the CONDOR distributed parallel computation infrastructure. SA searches used Matlab's built in simulated annealing function (<http://www.mathworks.com/help/gads/how-simulated-annealing-works.html>) with a maximum of 200 iterations (adding iterations did not significantly improve the best or average score of the final media). SA searches were run with an exponentially decreasing temperature function: $T\left( t+1 \right)=T\left( t \right)\cdot0.95$ (this function delivered the best results, i.e., the lowest p-value found).

A custom acceptance algorithm was used – given the current point with score $S1$, a new point with score $S2$ would be accepted if $S2<S1$ or if $S2\geq S1$with probability $1/\left( 1+e^{\frac{\Delta}{T}} \right)$. $T$ is the current temperature and $\Delta=\frac{S2-S1}{Scale}$ where $Scale$ was set to 0.05 representing the scale of significant p-values.

A custom annealing algorithm was used – given the current point $\boldsymbol{p}1$ which is a binary vector of length $n$, the new point $\boldsymbol{p}2$ was determined as follows:

1. Rnd<= rand();
2. if (Rnd<JumpThreshold)
3. Toggle <= JumpSize;
4. else
5. Toggle <= max(ceil(($n$/4)*($T$/100)),ceil($n$/100));
6. end
7. do until p2 is viable
8. p2<= p1 with Toggle location flipped
9. end

Where $T$ is the current temperature, JumpThreshold$=0.01$ and JumpSize $= \left\lceil n/8 \right\rceil$. The algorithm flips a certain temperature dependent number of locations in $\boldsymbol{p}1$ while keeping the number not too large when $T$ is high and not too small when $T$ is low (line 5). It also performs a large jump from time to time (line1-3). If the new point is not viable, another point is generated. An environment was said to be viable if the result of an FBA run for biomass maximization was above some small threshold [6], [7]. For each model, a small set of intake reactions was determined to be mandatory for biomass production (such as Zn, Mn, Mg and K intake reactions). These were kept open throughout the SA search since any media not containing them would not have been viable.

The purification stage was built of cycles: in each cycle open intake reactions were closed one by one and remained closed if the medium without them was viable and induced an essential gene set which kept the current KOR score, or improved it. After all intake reactions were tested, another cycle would commence until a cycle where no intake reactions were successfully shut down, was reached.

A typical ECOEDS run on ~70 models, with 250 repetitions on each model and 200 iterations in the SA search, took around 18 hours. Although only 200 essential gene computations are needed in the SA stage, the Purification stage may require several hundreds more, depending on the number of cycles and the number of open intakes. We estimate that over 8,000,000 whole metabolic model genome essential gene calculations were done on such a run.

For all models, the unique set of media from the final 250 media was always smaller than 250, despite the algorithm’s random start and despite the random nature of both the SA and the Purification stages, This means that some searches converged to the same result and the attribute Media Multiplicity Factor relates to this (S1 sheet "Biological and Model Scores").

## ECOEDS efficacy and convergence

The study results are based on 250 ECOEDS and 250 anti-ECOEDS runs performed on each model. It is likely that these provide media with the optimal KOR score or very close to it for the following reasons: (1) the best KOR score improved only slightly when runs were added as shown in Fig5. (2) For all models, running ECOEDS 250 times resulted in 250 media which were never all unique. This means that the algorithm converges to local minima. As the number of runs increase, the number of resulting unique results grows slower than the number of runs as shown in Fig4. (3) ECOEDS is shown to find media with significantly better KOR scores than random media (Fig6) and synthetic media (manuscript Results 3.3, Fig4).

Fig4 – ECOEDS Media Multiplicity convergence


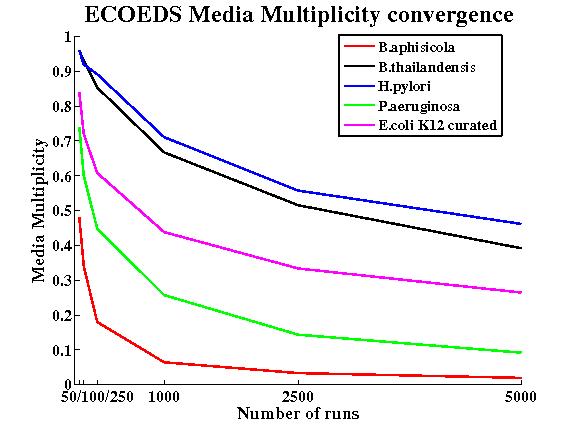


Six tests were done, making 50,100,250,1000,2500 and 5000 runs of ECOEDS respectively. The Media Multiplicity is defined to be the number of unique media divided by the number or runs.

Fig5 – ECOEDS KOR score convergence


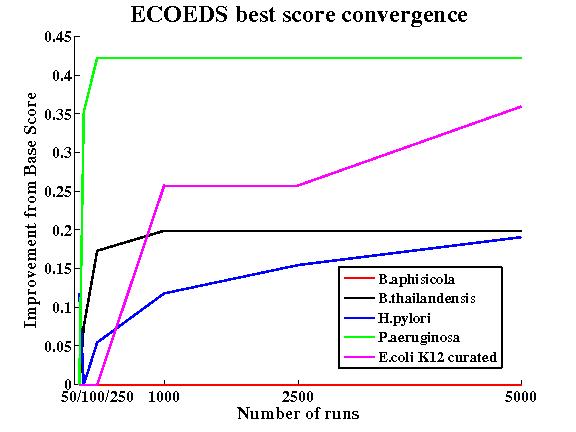


Six tests were done, making 50,100,250,1000,2500 and 5000 runs of ECOEDS respectively. For each test, the best KOR score found is shown. The Y-axis shows the incremental improvement in the score, i.e. scores are normalized by the lowest score found over all six tests. This procedure was repeated for 6 metabolic models as detailed in the figure legend. *B. aphisicola* did not benefit at all from more ECOEDS search iterations and the other SEED models seem to level out after less than 1000 runs. The curated *E. coli* iJO1366 model, probably due to its complexity, may still have room for improvement, even after 5000 runs.

Fig6 – Random media and ECOEDS media KOR scores


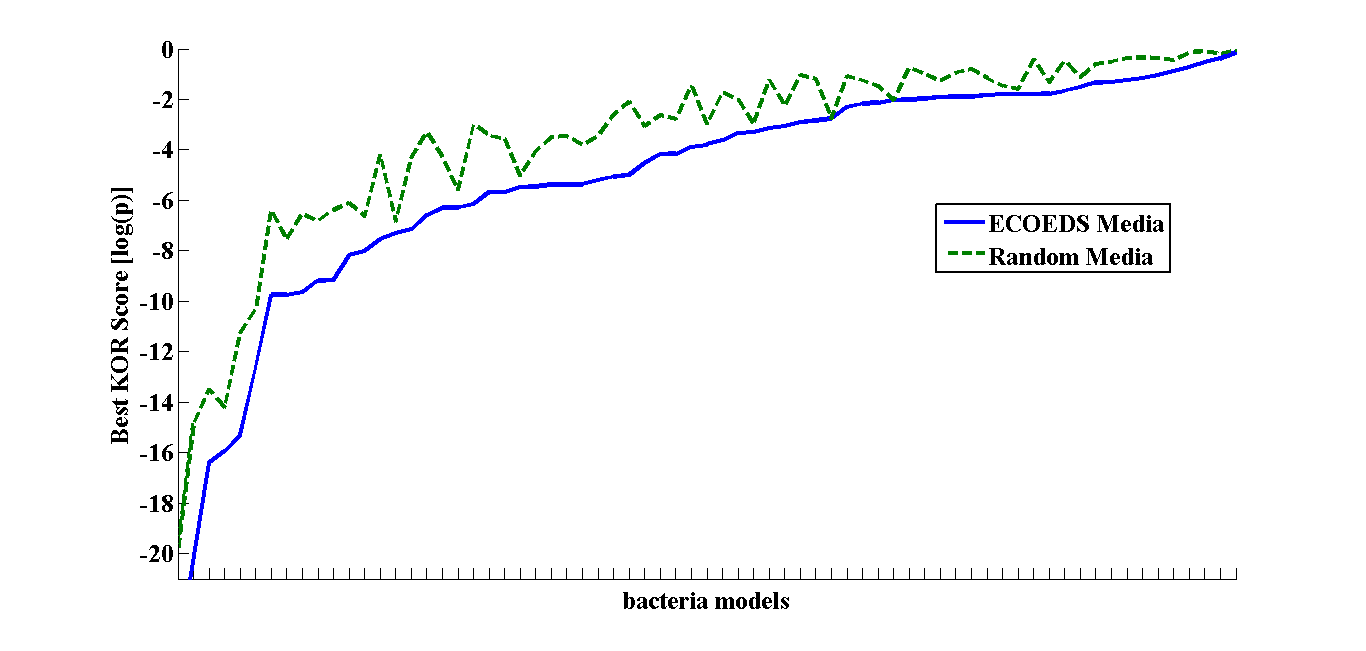


A set of viable Random Media was obtained for each model and their KOR score calculated. For each model, the best KOR scoring random medium is shown compared to the best ECOEDS medium. ECOEDS media always scored better then random media, except for 2 cases, where the scores were even (for the *R. prowazekii* and *U. urealyticum* models). The fact that 67 of 69 cases showed an improvement, testifies that ECOEDS offers a real optimization, consistently finding media with lower KOR scores. Plot is arranged to display scores in increasing order.

## Metabolic-model processing

This section provides details on the metabolic models used in this study – their selection criteria, model origin and model processing (Fig7).

### Model types

Two types of metabolic models were used in this study: automatically generated and human curated. Automatically generated SEED models[8] were downloaded from <http://seed-viewer.theseed.org/> and the human curated models were gathered form online resources; the full list of models and their references can be found in S1 sheet "Models DB". Some of the human curated models were given in SBML format and libSBML[9] was used to convert them to COBRA[10] format.

### Mapping dN/dS values to model genes

The ATGC database provides dN/dS estimates for bacteria via a list of NCBI gene IDs and a corresponding list of dN/dS values. NCBI IDs for each model genome were therefore necessary in order to map between model genes and ATGC dN/dS estimates. NCBI IDs for SEED model genes were taken from the .fig files accompanying each model. The .fig files contain a list of NCBI ID synonyms for each model gene. We then looked for a match between an ID from this list and an ID from the ATGC list of NCBI IDs for this organism. NCBI IDs for human curated model genes were found by (1) looking for the model gene name (e.g. KPN_0002 for *K. pneumonia* iYL1228) in the corresponding SEED model .fig file and then using the list of NCBI ID synonyms for this gene or (2) through .ptt files from the NCBI database <ftp://ftp.ncbi.nlm.nih.gov/genomes/Bacteria/>.

### Separation of Exchange Reactions

In order to separate intake from secretion in metabolic model FBA flux solutions, each exchange reaction was duplicated into separate "intake" and "secretion" reactions:

For "intake", the $S$-matrix coefficients were positive, with bounds

$$\text{lb}=0, \text{ub}=-\text{previous}\text{\_}\text{lb}$$

limiting the reaction to intake only.

For "secrete", the $S$-matrix coefficients were negative, with bounds

$$\text{lb}=0, \text{ub}=\text{previous}\text{\_}\text{ub}$$

limiting the reaction to secretion only.

### Random Media

A set of random media was computed for each model. The random media were used as seeds for ECOEDS SA search and as a comparison with ECOEDS results (Fig6). These media were sampled randomly from the metabolic model viable media space (biomass production above some predefined threshold). Similar to media processed in ECOEDS, random media were reduced to a binary version, i.e., each intake reaction is either open or closed (can/cannot carry flux). All bounds for reaction fluxes that were open were set to the same number (100), chosen to be high enough to surpass the (highest) positive lower bound some models place on certain constitutive reactions (such as ATP-production).

### Essential Gene Calculation Preprocessing

Model-based predictions of essential genes were calculated many times during this study. To speed up these calculation, preprocessing was done on all models to prepare data structures for quick calculations (S3 ‎2.2).

### Models Used In Study

In order for a model to be included in the study it needed to meet several criteria:

1. A Gene-Protein-Reaction (GPR) mapping must be present.
2. A biomass reaction must be present.
3. A non-zero maximum biomass must be attained on a rich medium.
4. A reference organism was found in the ATGC database to produce a genome-wide synonymous substitution rate estimate in the range of 0.25-1.5.
5. We were able to map dN/dS values to more than 50% of its genes.
6. Environmental information was present for the bacterium to allow the Biological Attribute analysis (manuscript Results 3.5).

All SEED models passed the first 3 criteria but surprisingly, there was a wide distribution regarding the percent of NCBI IDs that could be matched between the .fig files and the ATGC data (S1 sheet "SEED without NCBI matches"). Out of about 500 SEED organisms that passed criterion 6, only ~80 passed criterion 4. Out of the 40+ curated metabolic models gathered, ~25 passed the first 3 criteria and only 13 passed all 6 criteria. The final model list for this study includes 69 models consisting of 56 SEED models and 13 human curated models covering 58 bacterial species (S1 sheet “Models DB”).


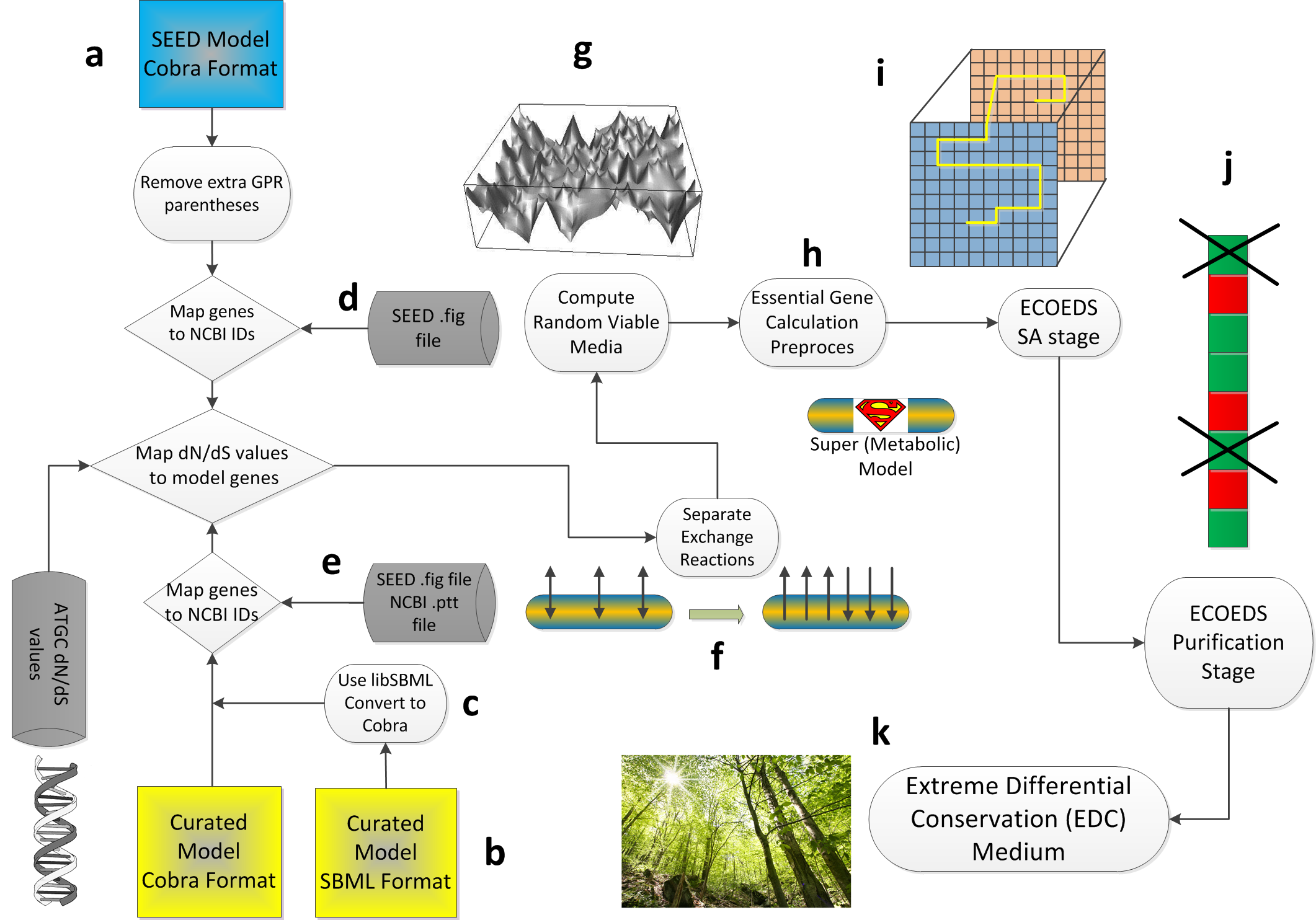


Fig7 – Metabolic model preparation pipeline

The pipeline was applied to models acquired from the SEED database (a) or from manually curated sources (b), which may needed translation to Cobra format (c). SEED .fig files and NCBI .ptt files were used to map model genes into unique NCBI IDs from the ATGC database (d,e). Model exchange reactions were separated (f) and random media calculated (g). Models were preprocessed for quick essential gene calculations (h) and then used in ECOEDS SA search (i) and Purification (j), the result of which was a set of EDC media (k).

## Correcting for multiple hypotheses

Testing for a connection between the KOR score and biological attributes, 36 different partitions of the 69 models into KOR and non-KOR groups were used. These tests are, however, highly dependent and so no correction for multiple hypotheses was done (even without correction no partition of values was found to be significant). Looking for a connection between KOR score and phylogenetic origin (S3‎1.5), each class and phyla were checked for enrichment in either KOR or non-KOR bacteria. The bacteria in the study cover 7 phyla and 10 classes and so using the Bonferroni method, we deemed a phylum significantly enriched if its Hypergeometric p-value was below ${0.01}/7$ and a class significantly enriched if its Hypergeometric p-value was below ${0.01}/{10}$. Looking for a connection between KOR score and habitat (S3 ‎1.6), each habitat or lifestyle were checked for enrichment in either KOR or non-KOR bacteria. Again, the Bonferroni method was used, each time accounting for the number of different environments.

Finally, the tests for a connection between – KOR score and biological attributes, KOR score and phylogenetic origins and KOR score and habitat – can be seen as different (and somewhat independent) hypotheses. Therefore, a factor-of-three multiple hypotheses correction was done across all experiments. This additional factor is discussed in S3 ‎1.5.

# Supplementary Information

S1 includes all information about the models in the study and the biological scores:

- Sheet "Model DB" Holds the list of bacteria and their model attributes.
- Sheet "Phylogeny" holds the phylogenic info for the bacteria in this study.
- Sheet "EGS KOR scores" holds the KOR scores according to Essential Gene Set splitting methods. It also holds the KOR classification of the significant scoring models.
- Sheet "Biological and Model Scores" includes all biological attributes used and their values for all models.
- Sheet “Essential Gene Sets” the sizes of the sets for all models, use for S3 Fig1.
- Sheet "HG Envs" holds a list of the environmental habitats from two databases [1], [2] and the number of models under each.
- Sheet "SEED without NCBI matches" includes a list of SEED models whose genes we were not able to pair up with enough NCBI IDs.
- Sheet "KOR classification" includes a list of all models and their corresponding KOR class.

S2 holds:

- Sheet “Biolog Attributes” holds a list of the biological attributes with information and references regarding each.
- Sheet “ECOEDS Convergence” holds the results of the convergence tests used for S3 Fig4 and S3 Fig5.
- Sheet “DEG Validation” holds the data for manuscript Fig3.

# Supplementary Section Bibliography

[1] S. Chaffron, H. Rehrauer, J. Pernthaler, and C. von Mering, “A global network of coexisting microbes from environmental and whole-genome sequence data.,” *Genome Res.*, vol. 20, no. 7, pp. 947–59, Jul. 2010.

[2] M. Parter, N. Kashtan, and U. Alon, “Environmental variability and modularity of bacterial metabolic networks.,” *BMC Evol. Biol.*, vol. 7, no. 1, p. 169, Jan. 2007.

[3] B. J. Huttenhower C, Gevers D, Knight R, Abubucker S, “Structure, function and diversity of the healthy human microbiome.,” *Nature*, vol. 486, no. 7402, pp. 207–14, Jun. 2012.

[4] P. S. Novichkov, I. Ratnere, Y. I. Wolf, E. V Koonin, and I. Dubchak, “ATGC: a database of orthologous genes from closely related prokaryotic genomes and a research platform for microevolution of prokaryotes.,” *Nucleic Acids Res.*, vol. 37, no. Database issue, pp. D448–54, Jan. 2009.

[5] T. Shlomi, M. N. Cabili, M. J. Herrgård, B. Ø. Palsson, and E. Ruppin, “Network-based prediction of human tissue-specific metabolism.,” *Nat. Biotechnol.*, vol. 26, no. 9, pp. 1003–10, Sep. 2008.

[6] A. Varma, B. W. Boesch, and B. O. Palsson, “Stoichiometric interpretation of Escherichia coli glucose catabolism under various oxygenation rates.,” *Appl. Environ. Microbiol.*, vol. 59, no. 8, pp. 2465–73, Aug. 1993.

[7] J. S. Edwards, R. U. Ibarra, and B. O. Palsson, “In silico predictions of Escherichia coli metabolic capabilities are consistent with experimental data.,” *Nat. Biotechnol.*, vol. 19, no. 2, pp. 125–30, Feb. 2001.

[8] C. S. Henry, M. DeJongh, A. A. Best, P. M. Frybarger, B. Linsay, and R. L. Stevens, “High-throughput generation, optimization and analysis of genome-scale metabolic models.,” *Nat. Biotechnol.*, vol. 28, no. 9, pp. 977–82, Sep. 2010.

[9] B. J. Bornstein, S. M. Keating, A. Jouraku, and M. Hucka, “LibSBML: an API library for SBML.,” *Bioinformatics*, vol. 24, no. 6, pp. 880–1, Mar. 2008.

[10] J. Schellenberger, R. Que, R. M. T. Fleming, I. Thiele, J. D. Orth, A. M. Feist, D. C. Zielinski, A. Bordbar, N. E. Lewis, S. Rahmanian, J. Kang, D. R. Hyduke, and B. Ø. Palsson, “Quantitative prediction of cellular metabolism with constraint-based models: the COBRA Toolbox v2.0.,” *Nat. Protoc.*, vol. 6, no. 9, pp. 1290–307, Sep. 2011.
